# Prevalence of Noncarious Cervical Lesions and Associated Factors After Orthodontic Treatment: A Long‐Term Follow‐Up

**DOI:** 10.1155/ijod/5560362

**Published:** 2026-07-29

**Authors:** Simone da Silva Luz, Renata Cristina Faria Ribeiro de Castro, Flávia Martão Flório

**Affiliations:** ^1^ Faculty of Dentistry, São Leopoldo Mandic, Campinas, São Paulo, Brazil, slmandic.edu.br; ^2^ Department of Orthodontics – USP, Postdoc in Public Health – UNICAMP, Campinas, São Paulo, Brazil; ^3^ Department of Collective Health, São Leopoldo Mandic, Campinas, São Paulo, Brazil, slmandic.edu.br

**Keywords:** corrective orthodontics, dental occlusion, fixed orthodontic appliances, noncarious cervical lesions

## Abstract

**Objective:**

The study aimed to evaluate the long‐term occurrence of noncarious cervical lesions (NCCLs) in adults previously treated with fixed orthodontic appliances, considering tooth type, gingival phenotype, and the quality and stability of orthodontic treatment outcomes.

**Methods:**

This observational study included 41 adult patients with complete orthodontic records. Data were collected at three time points: T1, before orthodontic treatment; T2, immediately after removal of fixed appliances and placement of retainers; and T3, during a long‐term follow‐up visit. At T1 and T2, information was obtained from orthodontic records, plaster models, and intraoral photographs. At T3, patients underwent clinical examination and standardized intraoral photographs. The presence and number of NCCLs were assessed from photographs at the three time points. The Peer Assessment Rating (PAR) index was used to evaluate treatment outcomes and stability. Gingival phenotype and lower fixed retainer status were assessed at T3. Friedman’s ANOVA was used for comparisons over time, and the Mann–Whitney test was used for comparisons according to retainer status and gingival phenotype, with a significance level of 5%.

**Results:**

The mean interval was 2.5 years between T1 and T2 and 6.5 years between T2 and T3. The PAR index decreased significantly from T1 to T2 and remained stable from T2 to T3. The prevalence of patients with NCCLs was 24.4% at T1, 29.3% at T2, and 39.0% at T3. The number of teeth with NCCLs did not differ significantly between T1 and T2 but was higher at T3 than at T1 (*p* = 0.020). Premolars and molars were the most affected teeth. Patients with a thick gingival phenotype had fewer teeth with NCCLs than those with a thin phenotype at T1 and T2, but this difference was not statistically significant at T3.

**Conclusions:**

In this sample of adults previously treated with fixed orthodontic appliances, the frequency of NCCLs was higher at long‐term follow‐up, while orthodontic treatment outcomes remained stable according to the PAR index. Premolars and molars were the most frequently affected teeth. The findings should be interpreted cautiously because behavioral, biological, and clinical factors related to NCCLs were not assessed longitudinally.

## 1. Introduction

Malocclusions are considered an important public health problem because they affect more than half of the world’s population and may compromise dental esthetics and quality of life [1]. In this context, fixed orthodontic treatment remains a widely used therapeutic option for the correction of different types of malocclusions [2]. The demand for orthodontic treatment among the adult population has increased substantially in recent years [3]. Because adults may have been exposed for longer periods to mechanical, biological, and behavioral factors associated with dental hard tissue loss, long‐term evaluation after orthodontic treatment may be clinically relevant [4–6]. At the same time, a high occurrence of noncarious cervical lesions (NCCLs) has been reported in adult populations [4, 7, 8]. NCCLs are multifactorial conditions characterized by the slow and irreversible loss of dental hard tissue at or near the cementoenamel junction, in the absence of dental caries [5, 9]. These lesions have been the subject of numerous studies due to their complex etiology and high prevalence [4, 8]. Although NCCLs have traditionally been associated with older individuals, their occurrence among younger and middle‐aged adults has also raised clinical concerns [4].

These lesions usually appear as smooth surfaces, rounded indentations, or wedge‐shaped depressions, with the depth varying from shallow to deep [9, 10]. In addition to lesion morphology, the characteristics of the surrounding periodontal tissues should also be considered when interpreting cervical defects. Gingival phenotype has been investigated because it may influence periodontal response, the risk of gingival recession, and the planning of restorative and periodontal procedures [11, 12]. Thin gingival tissues may be more susceptible to recession and root surface exposure, conditions frequently associated with NCCLs [4, 10, 13].

Occlusal stress has been proposed as one of the possible factors involved in the development of NCCLs [5, 14]. Malocclusion and unfavorable tooth positioning may contribute to nonaxial occlusal loading and stress concentration in the cervical region [15]. Nevertheless, the role of occlusal forces should be interpreted within a multifactorial framework. NCCLs may be influenced by the interaction of mechanical stress, friction, chemical degradation caused by intrinsic and extrinsic acids, toothbrushing practices, parafunctional habits, and cumulative exposure over time [4–6, 16–18]. Therefore, they should not be attributed to a single isolated factor.

In patients who have undergone orthodontic treatment, the long‐term stability of occlusal relationships is also relevant when interpreting cervical findings. Although the etiology of postorthodontic treatment relapse is not completely understood and cannot be predicted by isolated factors, it is widely accepted that it can occur even in treatments that have achieved good functional occlusion [19] and may be associated with postgrowth changes in the craniofacial complex [20]. Maintaining orthodontic treatment outcomes over time remains an important clinical challenge, particularly in the mandibular anterior region [21]. Preservation of the pretreatment dental arch form has therefore been recommended as one strategy for improving long‐term stability [22].

Although the multifactorial nature of NCCLs is widely recognized, the relative contribution of individual mechanical, chemical, and behavioral factors remains uncertain, and the available evidence is limited by methodological heterogeneity and the quality of existing studies [4, 16]. Systematic reviews have therefore emphasized the need for long‐term investigations using standardized assessment methods and adequate control of potential confounding factors [16–18].

Recent long‐term studies conducted in nonorthodontic populations have expanded the available evidence by evaluating NCCLs together with occlusal tooth wear, gingival recession, and behavioral exposures using three‐dimensional digital methods [23–25]. A 25‐year clinical follow‐up study found that the presence of NCCLs was associated with occlusal wear, occlusal interferences, acidic diet, and alcohol consumption [23]. A subsequent study documented a significant increase in occlusal tooth wear over the same follow‐up period [24], while a quantitative three‐dimensional analysis identified associations among NCCL dimensions, gingival recession, occlusal tooth wear, and several behavioral and clinical factors [25]. Together, these findings support the interpretation of NCCLs within a broader model of cumulative and interacting exposures.

Despite the availability of long‐term studies on NCCLs in nonorthodontic populations, the evidence remains limited regarding adults previously treated with fixed orthodontic appliances. A previous retrospective study evaluated the prevalence of NCCLs in orthodontically treated patients using clinical records and photographs [7]. However, it did not include a long‐term clinical reassessment of the participants. In particular, few studies have combined long‐term assessment of NCCL occurrence with tooth type, gingival phenotype, and objective measures of orthodontic treatment quality and stability. This distinction is important because orthodontic treatment may modify occlusal relationships, whereas the occurrence of NCCLs over time may also reflect cumulative exposure to age‐related, anatomical, mechanical, behavioral, and biological factors, including gingival phenotype and tooth type [4–6, 23–25].

To address this gap, the present observational study combined the retrospective assessment of existing orthodontic records at T1 and T2 with a long‐term clinical evaluation conducted specifically for the present study at T3. Therefore, this study aimed to evaluate the long‐term occurrence of NCCLs in adults previously treated with fixed orthodontic appliances, according to tooth type, gingival phenotype, and the quality and stability of orthodontic treatment outcomes.

## 2. Methodology

This observational study combined the retrospective assessment of existing orthodontic records at T1 and T2 with a long‐term clinical follow‐up examination conducted specifically for the present study at T3. The study was approved by the Institutional Ethics Committee (CAAE: 52215821.1.0000.53744). The records of adult patients who had completed orthodontic treatment were selected from orthodontic offices in Teresina, Brazil, without any influence from the treating dentist. All participants included in the long‐term follow‐up visit provided informed consent before the clinical examination at T3.

**Figure 1:**
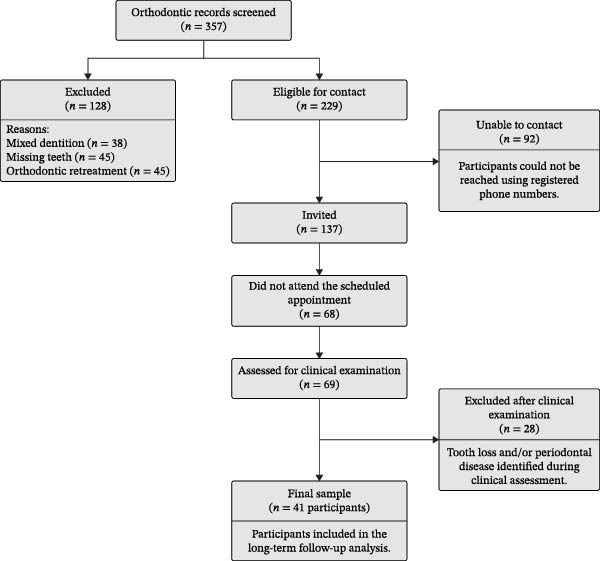
Flowchart of screening, eligibility, clinical assessment, and final sample inclusion.

The inclusion criteria considered participants with complete documentation of all previous orthodontic treatment appointments, including orthodontic models and intraoral, frontal, lateral, and occlusal photographs before and after treatment. Patients should have undergone fixed appliance orthodontic treatment, with a full permanent dentition from first molars to first molars at the beginning of the treatment. Additionally, participants should have had maxillary and mandibular retainers placed immediately after active orthodontic treatment and, at the time of selection for the study, should have completed orthodontic treatment at least 3 years prior. Patients undergoing orthodontic retreatment, those with a history of periodontal disease, as well as those with dental loss or agenesis, were excluded. The participant selection process and the final sample composition are presented in Figure 1.

**Figure 2:**
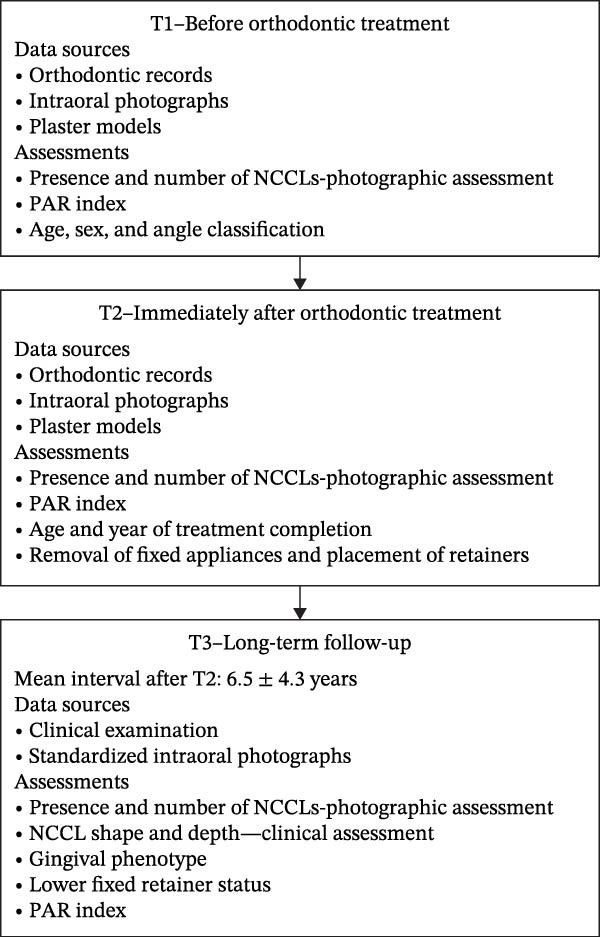
Chronological sequence of data sources and assessments performed at T1, T2, and T3.

Data for T1 and T2 were obtained retrospectively from existing patient records and orthodontic documentation, whereas T3 consisted of a long‐term clinical follow‐up examination conducted specifically for the present study. The three time points were defined as follows: T1 (baseline before orthodontic treatment), T2 (immediately after the completion of treatment, with the removal of upper and lower fixed appliances and the placement of retainers), and T3 (long‐term follow‐up visit). The chronological sequence of the data sources and assessments performed at T1, T2, and T3 is shown in Figure 2.

Treatment outcomes (difference between T1 and T2) and treatment stability (difference between T2 and T3) were evaluated using the Peer Assessment Rating (PAR) index on photographs and plaster models at T1 and T2 and clinically at T3. For NCCLs, the presence and number of affected teeth were assessed from photographs at three time points. The morphological characteristics and depth of NCCLs were clinically assessed only at T3 and were therefore not interpreted as directly comparable longitudinal measurements from T1 to T3. A trained and calibrated examiner conducted the photographic examinations. The interexaminer agreement Kappa coefficient was 0.85 for the number of NCCLs and 0.84 for the PAR index.

The analysis of medical records, photographs, and orthodontic models led to demographic and clinical information of patients collected at T1 (baseline before orthodontic treatment), including gender, age at the start of orthodontic treatment, angle malocclusion classification, presence and number of NCCLs, and PAR index. At T2 (immediately after treatment completion), information on orthodontic, clinical, and photographic aspects was gathered, including the year of completion, age at the end of treatment, presence and number of NCCLs, and PAR index. At the clinical examination conducted only at T3, the presence of lower orthodontic retainers, gingival phenotype, shape and severity of NCCLs, and static occlusion using the PAR index were clinically evaluated, and standardized intraoral photographs were taken for photographic assessment of the presence and number of NCCLs, following the same diagnostic criterion used for T1 and T2 photographs.

High‐resolution intraoral photographs (3008 × 2000 pixels) of both arches up to the first permanent molars were captured at T3 by the SSL operator using a Canon camera body (T6 Rebel), Canon Macro 105 mm USM lens, and twin flash with 1/64 power (Yongnuo Twin Macro YN14EX). Frontal photographs were taken perpendicular to the labial surface of the upper central incisors with a focal distance of 0.5 m, a diaphragmatic opening of 22, and a shutter speed of 1/125. Right and left lateral photographs were taken perpendicular to the buccal surface of the respective upper first premolars under the same parameters, except for the 25° diaphragm opening.

### 2.1. Evaluation of the Presence and Severity of NCCLs

Photographs taken at the three study time points were analyzed by two trained and calibrated orthodontists (Simone da Silva Luz and Renata Cristina Faria Ribeiro de Castro). Any mineral loss near the cementoenamel junction of the tooth not related to caries was considered NCCL [7, 26]. Each tooth was classified as having NCCL present or absent based on the photographs. For smaller lesions, the evaluation was based on a comparison with the morphology of adjacent and contralateral teeth [26]. Thus, longitudinal comparisons of NCCLs were restricted to the presence and number of affected teeth identified in photographs.

The morphological characteristics of the NCCLs were clinically evaluated at T3, considering the most severely affected tooth. The evaluation was conducted under artificial light, with drying, using a millimeter periodontal probe (Duflex, S. S. White, Rio de Janeiro, Brazil) and classified according to the following:

a. Shape (rounded, wedge‐shaped, or mixed), based on the angle formed at the pulpal wall of the lesion;
b. Depth (shallow, moderate, and deep) according to the cervical tooth wear index criteria [27].

The millimeter periodontal probe was placed perpendicular to the long axis of the tooth, at the center of the lesion, to determine the tooth wear index as follows: 0 = absence of NCCL; 1 = shallow NCCL with <1 mm depth; 2 = moderate NCCL with 1–2 mm depth; 3 = deep NCCL with >2 mm depth; and R = restoration, darkened surface, fracture, caries, or calculus. Because this clinical probing assessment was performed only at T3, lesion shape and depth were described only for the long‐term follow‐up examination.

### 2.2. Evaluation of Static Occlusion: PAR Index

The PAR index [28] was used to quantitatively evaluate intra‐arch and interarch occlusal relationships, as observed in plaster models at T1 and T2, and clinically at T3. The PAR index is calculated from 11 components: right upper segment, upper anterior segment, left upper segment, right lower segment, lower anterior segment, left lower segment, right posterior occlusion, left posterior occlusion, overjet, overbite, and midline. The percentage reduction in the PAR index score was considered in this study [28].

### 2.3. Evaluation of Gingival Phenotype

Gingival phenotype was assessed at T3 using the transparency of a periodontal probe through the gingival margin, a standardized dichotomous method supported by the scientific literature. If the outline of the probe could be visually detected through the tissue, the phenotype was categorized as thin; otherwise, it was categorized as thick [29, 30]. Interexaminer agreement was calculated for the number of NCCLs and the PAR index but not separately for gingival phenotype.

### 2.4. Orthodontic Retainers

The condition of the lower fixed retainer was evaluated, and any breakage or detachment was considered as a missing retainer.

### 2.5. Statistical Analysis

The collected data were recorded on standardized spreadsheets. The normality of the studied variables (PAR index and number of affected teeth) was assessed using the Kolmogorov–Smirnov test. Since normality was not observed at all time points (T1, T2, and T3), nonparametric tests were used. Thus, Friedman’s ANOVA (repeated measures) was used to evaluate the significance of differences over time (T1, T2, and T3), followed by multiple comparison tests to assess the significance of differences between T1 and T2, T2 and T3, and T1 and T3. For comparison between patients with and without retainers and between different gingival phenotypes, the Mann–Whitney test for independent samples was used, with a significance level of 5%. Statistical analysis was performed using the Statistical Package for the Social Sciences (SPSS) program, version 26 for Windows [31]. Because behavioral and biological factors such as toothbrushing habits, acidic diet, parafunctional habits, reflux, and salivary conditions were not systematically recorded at all time points, these variables were not included in the statistical models.

## 3. Results

The researcher screened 357 orthodontic records from orthodontic offices, of which 128 were excluded for not meeting the inclusion criteria (38 related to individuals with mixed dentition, 45 to individuals with missing teeth, and 45 to individuals undergoing retreatment). A total of 229 individuals were then selected, but 92 of them could not be contacted via their registered phone numbers. A total of 137 individuals were invited, but 68 could not attend the scheduled appointment. Of the 69 participants who showed up for the clinical examination, on average, 6.5 (± 4.3) years after the end of treatment, 28 met one or more exclusion criteria, including tooth loss and periodontal disease. Thus, 41 patients met the criteria for participation in the study. The participant flow is shown in Figure 1.

Table 1 presents the sociodemographic and clinical data of the participants, showing that the majority were female (*n* = 29; 70.7%), had Class II Angle malocclusion (*n* = 22; 53.7%), had a thick gingival phenotype (*n* = 28; 68.3%), and used lower retainers at T3 (*n* = 27; 65.9%). The mean age at the start of treatment and in the long‐term follow‐up was 21.9 (±8.2) and 31.0 (±8.0) years, respectively, and the active treatment duration was 2.5 (±1.4) years.

**Table 1:** Sample characterization and treatment time intervals (*n* = 41).

Variables		Descriptive measures
Sex	Female—*n* (%)	29 (70.7%)
	Male—*n* (%)	12 (29.3%)
Age		
At T1	Mean (standard deviation)	21.9 (8.2)
At T2	Mean (standard deviation)	24.5 (8.3)
At T3	Mean (standard deviation)	31.0 (8.0)
Time between treatments (years)		
Between T1 and T2	Mean (standard deviation)	2.5 (1.4)
Between T2 and T3	Mean (standard deviation)	6.5 (4.3)
Between T1 and T3	Mean (standard deviation)	9.0 (4.6)
Lower retainer at T3	No—*n* (%)	14 (34.1%)
	Yes—*n* (%)	27 (65.9%)
Gingival phenotype	Thin—*n* (%)	13 (31.7%)
	Thick—*n* (%)	28 (68.3%)
Angle malocclusion classification	Class I—*n* (%)	19 (46.3%)
	Class II—*n* (%)	22 (53.7%)

Table 2 presents the characterization and comparison of the PAR index between T1, T2, and T3 in the total sample, by retainer presence at T3, and by gingival phenotype. It can be observed that there was a significant reduction in the PAR index from T1 to T2 (−7.32 ± 5.06; *p* < 0.001) but not between T2 and T3 (0.37 ± 1.28; *p* = 0.581), indicating stability of the orthodontic treatment outcomes during the follow‐up period. This trend over time was similar for patients with and without a retainer at T3 and for those with thin and thick gingival phenotypes.

**Table 2:** Characterization and comparison of the PAR index between T1, T2, and T3 (*n* = 41).

Variable	T1	T2	T3	Differences		
				T1–T2	T2–T3	T1–T3
Total sample	8.54 (5.61)	1.22 (2.08)	1.59 (2.32)	−7.32 (5.06)	0.37 (1.28)	−6.95 (5.20)
*p*‐Value (time)		**p** < 0.001		**p** < 0.001	*p* = 0.581	**p** < 0.001
By lower retainer at T3						
No	8.71 (6.01)	1.64 (2.44)	2.57 (2.82)	−7.07 (5.50)	0.93 (2.06)	−6.14 (5.83)
*p*‐Value (time)		**p** < 0.001		**p** < 0.001	*p* = 0.450	**p** = 0.006
Yes	8.44 (5.51)	1.00 (1.88)	1.07 (1.88)	−7.44 (4.92)	0.07 (0.38)	−7.37 (4.91)
* p*‐Value (time)	**p** < 0.001			**p** < 0.001	*p* = 0.892	**p** < 0.001
* p*‐Value (retainer comparison)	*p* = 0.879	*p* = 0.361	**p** < 0.050			
By gingival phenotype						
Thin	5.62 (3.78)	1.08 (2.29)	1.38 (2.40)	−4.54 (2.82)	0.31 (1.11)	−4.23 (2.86)
* p*‐Value (time)		**p** < 0.001		**p** < 0.001	*p* = 0.845	**p** < 0.001
Thick	9.89 (5.86)	1.29 (2.02)	1.68 (2.33)	−8.61 (5.38)	0.39 (1.37)	−8.21 (5.59)
* p*‐Value (time)		**p** < 0.001		**p** < 0.001	*p* = 0.593	**p** < 0.001
*p*‐Value (phenotype comparison)	**p** = 0.026	*p* = 0.435	*p* = 0.430			

*Note:* Bold values indicate statistical significance (*p* < 0.05).

Table 3 shows that the proportion of patients with NCCLs was 24.4% (*n* = 10) at T1, 29.3% (*n* = 12) at T2, and 39.0% (*n* = 16) at T3, corresponding to an increase of 14.6 percentage points between T1 and T3. Although 10 individuals presented NCCLs at T1, the distribution of the most severely affected teeth included 11 teeth because one participant had two lesions in distinct teeth, both classified with the same severity. Premolars were the teeth most frequently classified as the most severely affected at all three time points, followed by molars. At T3, when lesion morphology and depth were clinically assessed, NCCLs were mostly wedge‐shaped and shallow.

**Table 3:** Characterization of NCCLs according to the most severely affected tooth at T1, T2, and T3 and lesion form and depth at T3.

Variable	T1		T2		T3	
Patients with NCCLs, *n* (%)	10 (24.4)		12 (29.3)		16 (39.0)	
Most severely affected tooth						
Worst affected tooth	*n*	%	*n*	*%*	*n*	%
Canines	0	0.0	0	0.0	1	6.3
Premolars	7	63.6	9	75.0	8	50.0
Molars	4	36.4	3	25.0	7	43.8
NCCL form						
Mixed	—		—		3 (18.8%)	
Wedge‐shaped	—		—		7 (43.8%)	
Rounded	—		—		6 (37.5%)	
NCCL depth						
Shallow	—		—		15 (93.8%)	
Moderate	—		—		1 (6.3%)	

Table 4 shows that there was no significant increase in the number of teeth with NCCLs during the active treatment period (T2–T1: 0.12 ± 0.40; *p* = 0.581). However, the number of teeth with NCCLs was significantly higher at T3 than at T1 (T3–T1: 0.59 ± 1.05; *p* = 0.020) and at T3 than at T2 (T3–T2: 0.46 ± 0.90; *p* = 0.040). The presence of a lower fixed retainer was not associated with the number of teeth with NCCLs (*p* > 0.05). Regarding gingival phenotype, patients with a thin phenotype had a higher mean number of teeth with NCCLs than those with a thick phenotype at T1 (*p* = 0.014) and T2 (*p* = 0.017), but this difference was not statistically significant at T3 (*p* = 0.082).

**Table 4:** Characterization and comparison of the number of teeth with NCCLs between T1, T2, and T3, considering mean (standard deviation) and percentage change (*n* = 41).

Variable	T1	T2	T3	Differences		
				T2–T1	T3–T1	T3–T2
Total sample	0.54 (1.29)	0.66 (1.35)	1.12 (1.81)	0.12 (0.40)	0.59 (1.05)	0.46 (0.90)
*p*‐Value (time)		**p** < 0.001		*p* = 0.581	**p** = 0.020	**p** = 0.040
				**22.2%**	**107.4%**	**69.7%**
By lower retainer at T3						
No	0.21 (0.58)	0.36 (0.63)	1.14 (1.92)	0.15 (0.36)	0.93 (1.54)	0.79 (1.31)
* p*‐Value (time)		**p** = 0.010		*p* = 0.705	*p* = 0.156	*p* = 0.108
				**71.4%**	**442.9%**	**216.7%**
Yes	0.70 (1.51)	0.81 (1.59)	1.11 (1.78)	0.11 (0.42)	0.41 (0.64)	0.30 (0.54)
* p*‐Value (time)	**p** = 0.001			*p* = 0.683	*p* = 0.066	*p* = 0.153
				**15.7%**	**58.6%**	**37.0%**
* p*‐Value (retainer comparison)	*p* = 0.257	*p* = 0.644	*p* = 0.888			
By gingival phenotype						
Thin	1.31 (2.02)	1.38 (1.98)	1.92 (2.25)	0.07 (0.28)	0.62 (0.96)	0.54 (0.78)
* p*‐Value (time)		**p** = 0.009		*p* = 0.845	*p* = 0.350	*p* = 0.170
				**5.3%**	**46.6%**	**39.1%**
Thick	0.18 (0.48)	0.32 (0.77)	0.75 (1.46)	0.14 (0.45)	0.57 (1.10)	0.43 (0.96)
* p*‐Value (time)		**p** = 0.001		*p* = 0.593	*p* = 0.082	*p* = 0.181
				**77.8%**	**316.7%**	**134.4%**
* p*‐Value (phenotype comparison)	**p** = 0.014	**p** = 0.017	*p* = 0.082			

*Note*: Percentage values indicate the relative change in the mean number of teeth with NCCLs for each comparison. Bold values indicate statistical significance (*p* < 0.05).

## 4. Discussion

Since the etiology and contributing factors to the initiation and progression of NCCLs during clinical interventions are not fully understood, these lesions are a concern for any elective dental treatment, as they are irreversible [9]. In the present study, the proportion of patients and the number of teeth with NCCLs were higher at the long‐term follow‐up visit than before orthodontic treatment, whereas no significant difference was observed during the active orthodontic treatment period. Premolars were the most frequently affected teeth, followed by molars. These findings are consistent with previous evidence showing a high occurrence of NCCLs in premolars and support the interpretation of these lesions as chronic multifactorial conditions influenced by cumulative mechanical, biological, and behavioral exposures [7, 9, 23–26].

### 4.1. Types of Teeth

Premolars have a smaller crown volume and, like molars, are exposed to substantial masticatory loads [32]. Their anatomical and biomechanical characteristics may favor stress concentration in the cervical region during eccentric mandibular movements, particularly during group function [6, 32, 33]. It has been demonstrated that, in premolars subjected to occlusal tensile forces, stress may become concentrated in the cervical region and within preexisting lesions, potentially contributing to continued dental hard tissue loss [33]. These findings, together with previous evidence of a higher prevalence of NCCLs in premolars, may help explain why premolars were the most frequently affected teeth in the present sample [34]. However, because lesion depth and morphology were clinically assessed only at T3, these characteristics describe the lesions at long‐term follow‐up and cannot be interpreted as evidence of longitudinal progression.

Molars were the second most frequently affected tooth group. As posterior teeth, molars are exposed to substantial and recurrent masticatory loads. Evidence from finite element analyses indicates that nonaxial occlusal loading may generate substantially higher cervical stress than axial loading [9]. Such loading patterns may increase stress concentration at the cementoenamel junction and contribute to cervical hard tissue deformation. In addition, molar morphology, including the proximity of the furcation to the cervical region, may influence stress distribution and contribute to cervical stress concentration [27]. Nevertheless, the distribution of NCCLs among molars cannot be explained by occlusal and anatomical factors alone. This interpretation should remain cautious because dynamic occlusion, occlusal interferences, and relevant behavioral and biological exposures were not assessed longitudinally in the present study.

### 4.2. Orthodontic Treatment

Current evidence does not establish a direct effect of orthodontic treatment on the incidence or progression of NCCLs, despite the temporary changes in force distribution associated with orthodontic tooth movement [9, 17]. It has been suggested that untreated malocclusion and unfavorable tooth positioning, such as excessive lingual inclination of the maxillary premolars, may contribute to cervical stress concentration, rather than orthodontic treatment itself, as indicated by finite element analysis [15]. This interpretation is consistent with the present findings, since the number of teeth with NCCLs did not differ significantly between T1 and T2, corresponding to the active orthodontic treatment period.

In contrast, a cross‐sectional study of 43 male athletes reported a positive association between previous orthodontic treatment and the number of NCCLs [35]. However, the participants had not necessarily completed the treatment, and the cross‐sectional design did not allow temporal relationships to be established. Taken together, these findings suggest that NCCLs should not be attributed exclusively to orthodontic treatment. Instead, their occurrence is likely to reflect interactions among occlusal conditions, tooth morphology, age, gingival phenotype, oral habits, dietary exposures, and other individual biological and behavioral factors [4–6, 23–25].

### 4.3. Age

The prevalence of NCCLs has been reported to increase with age [7, 36], and the participants in the present study had a mean age of 31 years at T3. Increasing age is accompanied by longer cumulative exposure to factors potentially associated with NCCLs, including cervical stress concentration, mechanical friction, and chemical degradation caused by intrinsic and extrinsic acids [5, 37]. Gingival recession and cervical surface defects are also clinically relevant in the assessment of exposed root surfaces [38], potentially exposing dentine, which may be more vulnerable than enamel to erosive degradation [39].

Long‐term studies [23–25] support this cumulative interpretation by showing that NCCLs, occlusal tooth wear, gingival recession, and behavioral and clinical exposures may interact over extended follow‐up periods. Although age was not analyzed as an independent associated factor in the present study, the higher frequency of NCCLs at T3 may partly reflect cumulative exposure over time rather than an isolated effect of orthodontic treatment.

### 4.4. Orthodontic Retainers

The orthodontic literature emphasizes the importance of maintaining lower fixed retainers because posttreatment relapse may occur even after satisfactory occlusion has been achieved [40, 41]. In addition, age‐related occlusal changes may occur even in individuals with normal occlusion, reinforcing that long‐term occlusal stability should be interpreted as a dynamic condition rather than a fixed endpoint [42]. In the present study, some participants returned for long‐term follow‐up without a lower fixed retainer. At T3, patients without a retainer had higher PAR index values than those with a retainer, although the between‐group difference was at the threshold of statistical significance (*p* = 0.050). This finding should therefore be interpreted cautiously, but it is consistent with the role of retention in preserving the orthodontic alignment over time [40, 41].

The reasons for retainer absence were not recorded. Although hygiene difficulties related to biofilm accumulation around fixed retainers [43] and retainer breakage [44] have been described in the literature, these explanations cannot be confirmed for the present sample. The number of teeth with NCCLs did not differ according to retainer status at T3 (*p* = 0.888), suggesting that retainer presence was not associated with NCCL occurrence in this sample. Regardless of this finding, long‐term retention remains an important strategy for preserving dental alignment after orthodontic treatment [40, 41, 45].

### 4.5. Gingival Phenotype

Gingival phenotype reflects anatomical characteristics of the periodontal tissues that are influenced by genetic factors, tooth morphology, and tooth position, and these tissue characteristics may affect susceptibility to gingival recession and clinical responses to dental treatment [11, 12]. In the present study, participants classified as having a thick gingival phenotype at T3 had fewer teeth with NCCLs than those with a thin phenotype at T1 and T2. However, the between‐group difference was not statistically significant at T3. Previous studies have indicated that thin gingival tissues may be more susceptible to gingival recession [11], and NCCLs are frequently observed in association with exposed dentin and root surfaces [5, 10, 18, 27].

The attenuation of the difference between phenotypes over time (T1: *p* = 0.014; T2: *p* = 0.017; and T3: *p* = 0.082) suggests that gingival phenotype alone may not fully explain NCCL occurrence at long‐term follow‐up. Cumulative mechanical, chemical, biological, and behavioral exposures may become increasingly relevant over time [4, 37, 46]. However, these factors were not assessed longitudinally in the present study, and this interpretation should therefore be considered cautiously. In addition, because gingival phenotype was assessed only at T3, its temporal relationship with NCCL occurrence cannot be established.

### 4.6. Other Variables

Behavioral and biological factors, including parafunctional habits such as bruxism, acidic dietary exposure, gastroesophageal reflux, salivary flow and composition, and oral hygiene practices, have been reported as relevant indicators associated with NCCLs [4, 5, 37, 46]. Long‐term clinical studies have further identified associations of NCCLs, gingival recession, and occlusal tooth wear with occlusal wear and interferences, acidic diet, alcohol consumption, smoking, vigorous toothbrushing, and other behavioral and clinical exposures [23–25]. These variables were not systematically recorded in the available records and, therefore, could not be evaluated as potential confounding factors in the present study. Future longitudinal investigations should collect these exposures using standardized and validated instruments, enabling more comprehensive adjustment for confounding and a more robust assessment of their temporal relationships with NCCL occurrence.

### 4.7. Limitations

First, the presence and number of NCCLs were assessed from photographs at T1, T2, and T3, whereas the clinical assessment of lesion morphology and depth was performed only at T3. Therefore, T3 clinical measurements of shape and depth should not be interpreted as directly comparable longitudinal measurements from baseline. Although the same diagnostic criteria were applied to photographs from all three time points, differences in image acquisition, angulation, lighting, and resolution inherent to retrospective records may have affected lesion detection and introduced assessment bias. Future studies incorporating standardized clinical and photographic examinations at all time points may reduce assessment bias and improve lesion characterization.

Second, the retrospective design limited control over data completeness and restricted the evaluation of relevant confounding variables, such as dietary acid exposure, parafunctional habits, gastroesophageal reflux, salivary factors, oral hygiene practices, and gingival recession. In addition, gingival phenotype was assessed only at T3; consequently, its temporal relationship with NCCL occurrence at T1 and T2 cannot be established.

Third, the absence of an untreated comparison group with similar baseline malocclusions limited the ability to distinguish the potential contribution of orthodontic treatment from the effects of aging and other cumulative exposures. Therefore, the present findings should not be interpreted as demonstrating a causal relationship between orthodontic treatment and NCCL occurrence. Prospective cohort studies including appropriate comparison groups and standardized longitudinal clinical assessments are warranted.

The participant selection process may also have introduced selection bias, because a substantial proportion of potentially eligible individuals could not be contacted, did not attend the scheduled examination, or were excluded at T3. Participants who completed the long‐term follow‐up may therefore differ from those who were not assessed. Finally, the sample size (*n* = 41) may have limited statistical power, particularly in subgroup analyses, and may restrict the generalizability of the findings. However, assembling long‐term follow‐up samples with complete pretreatment and posttreatment orthodontic documentation remains challenging.

Despite these limitations, this study provides long‐term follow‐up evidence on NCCLs in adults previously treated with fixed orthodontic appliances, integrating tooth type, gingival phenotype, and orthodontic treatment stability assessed using the PAR index. By assessing the presence and number of NCCLs photographically at three time points and complementing these data with clinical examination at long‐term follow‐up, the study contributes to a cautious interpretation of NCCLs as chronic multifactorial conditions whose occurrence cannot be explained solely by orthodontic treatment quality and stability.

## 5. Conclusions

• Orthodontic treatment quality improved significantly from T1 to T2, as shown by the reduction in PAR index values from 8.54 to 1.22 (*p* < 0.001), and remained stable at T3 (*p* = 0.581).
• The proportion of patients with NCCLs was higher at T3 than at T1, increasing from 24.4% to 39.0%, and the mean number of affected teeth was significantly higher from T1 to T3 (0.54 to 1.12; *p* = 0.020).
• At T3, premolars and molars accounted for 50.0% and 43.8% of the most severely affected teeth, respectively.
• Gingival phenotype was associated with the number of teeth affected by NCCLs at T1 (*p* = 0.014) and T2 (*p* = 0.017) but not at T3 (*p* = 0.082).
• Lower fixed retainer presence at T3 was not associated with the number of teeth affected by NCCLs (*p* = 0.888).
• Overall, NCCLs should be interpreted as chronic multifactorial conditions that may be observed more frequently over time in adults. Gingival phenotype may contribute to clinical risk profiling, but NCCL occurrence should not be attributed to orthodontic treatment quality or retention status alone.

## Author Contributions

**Simone da Silva Luz**: conceptualization, methodology, investigation, data curation, formal analysis, writing – original draft, writing – review and editing. **Renata Cristina Faria Ribeiro de Castro**: conceptualization, methodology, supervision, validation, writing – review and editing. **Flávia Martão Flório**: conceptualization, methodology, supervision, formal analysis, validation, writing – review and editing.

## Funding

This study did not receive any specific funding.

## Disclosure

All authors have read and approved the final version of the manuscript. Simone da Silva Luz, as the corresponding author and manuscript guarantor, had full access to all of the data in this study and takes complete responsibility for the integrity of the data and the accuracy of the data analysis.

## Conflicts of Interest

The authors declare no conflicts of interest.

## Data Availability

The data that support the findings of this study are available from the corresponding author upon reasonable request. The data are not publicly available due to privacy and ethical restrictions.
